# Boosting the development of individual placement and support in Europe

**DOI:** 10.1017/S2045796022000129

**Published:** 2022-04-28

**Authors:** Hlynur Jónasson, Jaap van Weeghel, Débora Koatz, Gary Johnston, Ulrika Bejerholm, Angelo Fioritti

**Affiliations:** 1 Landspitali, Psychiatric Hospital, Reykjavik, Iceland; 2 Phrenos Center of Expertise, Da Costakade 45, Utrecht, The Netherlands; 3 Tranzo Scientific Center for Care and Wellfare, Tilburg School of Social and Behavioural Sciences, Tilburg University, Tilburg, The Netherlands; 4 Avedis Donabedian Research Institute, Barcelona, Spain; 5 Universitat Autònoma de Barcelona (UAB), Barcelona, Spain; 6 Social Finance, London, UK; 7 Department of Health Sciences, Lund University/Research and Development Department, Mental Health Services, Region Skåne, Sweden; 8 Department of Biomedical and Neuromotor Sciences, School of Hygiene and Preventive Medicine, University of Bologna, Bologna, Italy

## Introduction

Employment is a critical factor in the rehabilitation and recovery of people with severe mental health problems (SMHP). However, employment rates for this group are far lower than in the general population and well-known are the many barriers for people with SMHP to enter the labour market. The right to employment is recognised in the European Disability Strategy and the European Pillar of Social Rights (principle 17) and the United Nations Convention on the Rights of Persons with Disabilities (UNCRPD art. 27). There is a strong need for having access to effective programmes offering multi-faceted supports in regaining and maintaining competitive employment. Individual placement and support (IPS) is such a programme *par excellence.*

Among the several policies, practices and interventions implemented in the last decades IPS stands out for its effectiveness and feasibility, demonstrated by an impressive amount of empirical studies. Among 28 randomised controlled trials (RCTs) comparing IPS to traditional vocational services, all but one showed superior competitive employment rates for IPS. From the 1990s IPS has grown rapidly, initially mainly in the US, proving to be easier than other psychosocial intervention to be transferred into routine practice and to be transported across different mental health systems (Bond *et al*., 2020).

A strong driving force behind the expansion is the IPS learning collaborative, guided by the IPS Employment Center in New Hampshire (Becker *et al*., 2014). Established in 2002, this collaborative coordinates education, training, technical assistance, fidelity assessment, quality assurance and outcome monitoring, as well as regular communications through newsletters, bimonthly calls and annual meetings. The growing size of the European IPS professional community now generates the need to establish a similar IPS learning community, in close collaboration with American colleagues, with a view to facilitate the sharing of expertise and the exchange of experience with regard to the dissemination, implementation, and evaluation of IPS practices across Europe in all its diversity.

In this paper, we describe the development of IPS practice in Europe, and the establishment, objectives and intended activities of the IPS European Learning Community (ELC).

## Development of IPS in Europe

At the beginning of this century the European scientific community viewed IPS with scepticism, doubting the possibility of replicating the extremely good results coming from the US, relying more on the long tradition of vocational rehabilitation based on sheltered workshops, training centres, social enterprises and legislated quota systems (Fioritti *et al*., 2014). IPS was first introduced on a limited scale in six European countries (Bulgaria, Germany, Italy, Netherlands, Switzerland and UK) that participated in the EQOLISE study, a RCT aimed to test the effectiveness of IPS. EQOLISE replicated the excellent results of American studies, despite extensive differences in labour market regulations, and in the organisation and culture of mental health services. IPS proved to be superior to treatment as usual for the number of people entering the competitive market (55% *v*. 26%), the number of days and hours worked, and the amount of money earned. Differences were found also as to hospitalisations, drop-out from treatment, or overall psychopathology, though not statistically significant (Burns *et al*., 2007).

Following on the EQOLISE study, a growing number of countries started to implement and further evaluate IPS. IPS has also been recommended in European policy documents (OECD, 2015; The European Mental Health Action Plan, 2013–2020, 2015; EU Joint Action on Mental Health and Wellbeing, 2016). IPS programmes are currently found in Belgium, Czech Republic, Denmark, Finland, France, Germany, Iceland, Ireland, Italy, Netherlands, Norway, Spain, Sweden, Switzerland and UK. And to date, nine more RCTs on IPS have been conducted, in six European countries (Heslin *et al*., 2011; Hoffmann *et al*., 2012, 2014; Michon *et al*., 2014; Bejerholm *et al*., 2015, 2017; Viering *et al*., 2015; Reme *et al*., 2018; Christensen *et al*., 2019; Sveinsdottir *et al*., 2020; Hellström *et al*., 2021), all with results in favour of IPS.

However, in Europe as a whole the diffusion of IPS is still patchy. The degree of implementation of IPS varies, as do implementation barriers that each programme has to face. In each European country, the organisation of governmental services, funding of mental health and vocational services, labour legislations, disability policies, national history and culture have all shaped the evolution of IPS services on a system level. Other critical components are also evident at the regional and local organisation levels (Bergmark *et al*., 2018).

Given this diversity, each country has followed its own pathway to IPS development (Fioritti *et al*., 2014; IIMHL, 2019; Drake *et al*., 2020), but mostly through two main ways. The first one goes bottom-up, with pilot centres developing it and then sensitising other professionals, managers and decision-makers. The second way goes top-down, with leaders, stakeholders and policy-makers adopting it regionally or nationally and lobbying by international bodies.

In some countries of Northern Europe (Sweden, Netherlands, Norway, Iceland and UK), the dissemination of IPS has gradually become a matter of national policy. In these countries national investments are made with regard to mobilisation, implementation, training needs analysis, fidelity reviews, action plans and local technical support, training and, research all as a combined approach for quality assurance. In other countries, the dissemination of IPS is guided by regional policies adopted after successful local programmes, such as in Italy (Emilia-Romagna) and Spain (Catalonia, Tenerife Island) (Rodríguez Pulido *et al*., 2017; Hilarión *et al*., 2020). In yet other countries (Belgium, Czech Republic, France) IPS is only conducted in some single pioneering practices, and in a few countries IPS programmes are implemented mainly for research purposes (Germany and Switzerland). Finally, most of Central and Eastern European countries are still in a preliminary phase.

As a consequence, IPS practices are currently financed from various sources: health-related funding, social welfare, labour activation funding, funding by charities and foundations, government funding, research grants, or a mixture of these funding sources. In a number of countries, IPS is provided by mental health care organisations, as it was originally done in the US (Iceland, Italy, Netherlands, UK), in other countries by municipal administrations or by NPOs under their allowance (Denmark, Norway, Sweden). In all countries, IPS is conducted by employment specialists with various professional backgrounds: e.g. occupational therapists, professional educators, social workers, nurses, psychologists. In some countries the training needed to become an IPS employment specialist is offered by national certified bodies (Netherlands, UK) or universities (Sweden).

In most countries, quality assurance activities targeting IPS practices are carried out through fidelity reviews and outcome analysis. Some minor adaptations to the IPS model are made locally, to make the language and delivery approach more congruent with present health and social organisations. Factors that stimulated the growth of IPS were: the impact of local IPS research studies, local champions and dedicated national leaders, participating in the international IPS learning collaborative, fidelity monitoring and, in some countries, the rapid growth of long-term disability rolls. These factors, plus the international consensus that IPS is a programme based on strong scientific evidence, explain the spread of IPS outside the US, both in Europe and in other continents (Bond *et al*., 2020; Drake *et al*., 2020).

## Towards a European learning collaborative on IPS

In 2019 a few enthusiasts from various countries (Iceland, Italy, Netherlands, Spain, UK) came to consider to start an IPS ELC. After some preparatory online discussion meetings, other IPS practitioners from European countries were identified and contacted by using professional networks, literature search and snowballing. They organised three webinars spaced over 6 months, in which representatives from different countries presented the state of affairs of IPS practices in their countries. Ultimately the face-to-face conference took place in Reykjavik, Iceland, on 16–17 September 2021. For this meeting, an Erasmus application was awarded to partly fund the event and member attendance.

The primary goal of this meeting was to establish the IPS ELC with IPS leaders from as many countries as possible. A learning community is neither a scientific association nor an educational enterprise. Actually, it is a community of practice, a partnership among people who find it useful to learn from and with each other about a particular domain. Participants use each other's experience of practice as a learning resource. And they join forces in making sense of and addressing challenges they face individually or collectively (Wenger, *et al*., 2011).

The inaugural IPS ELC conference in Reykjavik was held to discuss common objectives and activities: first, to build the case to support further scaling of IPS across Europe; second, to create resources and support for that scaling; third, to develop a learning community to guide IPS practice in the European context.

In total 48 individuals from thirteen countries participated in this conference. The following topics were addressed extensively by means of presentations, workshops and planning meetings: valuing and using personal experiences in IPS practice; fidelity, challenges and chances for programme evaluation and quality improvement; peer support/co production of IPS services; managing IPS and job retention; IPS and substance misuse; IPS and sharing personal information (disclosure). The group also explored how to best harness the talents, passion and expertise of the participants to best support the scaling of IPS across Europe. It was agreed upon that the ELC will continue to organise webinars every three months. And a face-to-face meeting will be organised every year.

## Reflections and future directions

What is needed is to support the completion of the process all over Europe. How can a ELC Community help to bring IPS into routine use?

To answer this question Drake and Wallach (2020) made a convincing argument that employment should above all be seen as a critical *health* intervention, based on five reasons. First, people with disabilities and those without disabilities deteriorate when they become unemployed. Second, people with disabilities and those without disabilities improve their mental health and wellbeing when they become employed (Harnois and Gabriel, 2000; Van Rijn *et al*., 2016). Third, we now have an effective intervention, IPS, that helps 40% to 60% of people with a wide range of health and psychosocial conditions to become competitively employed. Fourth, beyond the health benefits, we know that providing more good workers in competitive jobs helps businesses, the economy and the welfare system. Finally, helping people with disabilities to achieve a meaningful life is a moral imperative, a disability rights issue and a human rights issue.

The Helsinki Mental Health declaration (EUR/RC55/R2) in 2005 and the WHO European Mental Health Action Plan (2013–2020) made mental health a priority on the European Agenda, aiming at guaranteeing rights and integrating mental health evidence-based policies for opportunities associated with full citizenship including social affairs, education and employment. IPS can be very helpful in reaching these objectives, especially considering the rapid evolution it had in a wide range of directions.

First, there are many innovative IPS practices in European countries (Van Weeghel *et al*., 2020). IPS gets more and more extended to all individuals with mental illnesses. Initially, like in the US, IPS practice was limited to people with SMHP. Nowadays, for instance in the Netherlands, IPS practices are not only present in community mental health teams, but have also been developed successfully in psychiatric community housing programmes (De Winter *et al*., 2020). In Norway and Sweden, IPS has been made accessible to clients with common mental disorders and substance use disorders (Bejerholm *et al*., 2017; Reme *et al*., 2018; Hellström *et al*., 2021).

Second, there is an increasing variety of IPS providers. Originally, mental healthcare organisations have been the main actors adopting IPS practices. To attain optimal adoption and sustainability, now systems tend to form broad coalitions and shared ownership of IPS, including at both local and national levels, mental healthcare providers, general social services and employment services, the vocational rehabilitation system, service users and family organisations and (in some countries) health insurance companies (Bond *et al*., 2020). In some European countries, IPS is mainly provided by (contracting organisations of) municipal social service departments (e.g. in Denmark, Norway and Sweden). These departments are responsible for the vocational reintegration of the citizens that receive welfare benefits, who often have multiple problems, including mental health problems, and may benefit from the integrated approach that the IPS model offers. This development also raises the problem of how to ensure increased access to IPS without compromising fidelity (Bond *et al*., 2020).

Third, there is also an expansion of IPS objectives, e.g. education. Many young people with mental illness want to resume their education, having been compelled to suspend it during previous periods of illness. IPS's two-pronged approach (education and employment) is now mainly being put into practice in the treatment of clients with first-episode psychosis, but this must be extended to people with other mental illnesses.

Fourth, there is also a development in the use of additional interventions to reinforce the effects of IPS for clients. Examples of add-on interventions are workplace fundamentals (Glynn *et al*., 2017), cognitive remediation (McGurk *et al*., 2015; Christensen *et al*., 2019; Van Duin *et al*., 2021), Wellness Recovery Action Planning (WRAP) (Van Erp *et al*., 2015) and COnceal or ReveAL (CORAL), a decision aid for disclosure of mental health problems in the workplace (Henderson *et al*., 2013; Janssens *et al*., 2020).

Last, greater input is coming from companies and employers. Although IPS could not exist without the active involvement of employers, as yet they have not had a clear role or position in the dissemination of IPS (Van Weeghel *et al*., 2020). Nevertheless, ever more employers show an interest in ‘inclusive employment’, i.e. in a company where the work is organised in such a way that everyone is able to participate fully and to contribute to the operating result (Zijlstra *et al*., 2012).

This list of innovative elements in the implementation of IPS in Europe (which are also visible in the US) is not exhaustive. Other developments such as sharing personal experiences, peer-support workers in IPS programmes, and the use of virtual reality interventions should also be mentioned here. These innovations indicate the need for updating manuals and to write a European Manual leaded by the ELC.

## Conclusions

IPS is becoming more and more a full-scale strategy to attain inclusive and sustainable employment for people with serious health and psychosocial problems. We need to advocate jointly at international, national, regional and local bodies to promote IPS as the first-choice method for all public, private and NPOs employment services.

IPS balances economic and psychological elements by providing specific supports to help clients make rational choices regarding job seeking, combining personal goals, previous working experiences and objective opportunities either to work, to study, or to obtain a disability benefit. This approach has definitely proved beneficial to people with serious mental disorders, but it may also work for the general unemployed population, possibly with some adjustments. Employment services in Europe have had serious difficulties with the long-term unemployed population, people who lost jobs in their 50s, people with lower degrees of specialisation, migrants, single women with children and gender violence victims. IPS has all the ingredients to be considered a full-scale ‘active labour policy’ (Rizza and Fioritti, 2020) economically sustainable in this Covid-19 era, where the links between work, mental health and welfare policies is stronger than ever.

To attain this, good coordination and the integration of services of mental health care, social care and the vocational rehabilitation system is needed (OECD, 2015; Bergmark *et al*., 2019). The aims of such service integration are to improve access to an array of services, improve continuity of care, reduce the overlap, inefficiency and cost of services and to increase the involvement and responsibility of specialists (Konrad, 1996). IPS has proven to be successful in realising such coordination and service integration.

The IPS model is ‘simple but not easy’. Although it is not based on grand theory and is rather pragmatic in its approach, it requires strong commitment to be put into practice. Therefore we need to recognise critical implementation components and take stock over contextual barriers and facilitators. Strong local leadership will be the key to success for IPS for a long time forward. In spite of its simple theory, practice and training, IPS creates a cultural revolution within existing services that requires support and promotion by leading professionals.

Establishing through the ELC stable collaborations and exchanges by centres that actively practice IPS will enhance further development in Europe, improve sustainability and implementation in a European context, and allow testing its effectiveness in other target groups.
